# Supplementary material for: Controlling the nonadiabatic electron-transfer reaction rate through molecular-vibration polaritons in the ultrastrong coupling regime
Source: Sci Rep. 2020 Apr 30;10:7318. doi: 10.1038/s41598-020-62899-8 (PMC7193605; doi:10.1038/s41598-020-62899-8)
Supplement: Supplementary file 1 — Supplementary Information. [file 41598_2020_62899_MOESM1_ESM.pdf]

Supplementary Information for "Controlling the electron-transfer reaction rate through molecular-vibration polaritons in the ultrastrong coupling regime"

I. SECOND-ORDER PERTURBATION THEORY

In this section, we use the second-order perturbation theory to show that the coefficients c_0 and c_2 in the expansions [Eqs. (10) and (11) in the main text] have opposite signs.

We first derive the general expressions for the second-order perturbation theory by decomposing the Hamiltonian as

$$\hat{H} = \hat{H}_0 + \lambda \hat{H}_1, \tag{1}$$

where $\hat{H}_0$ is the unperturbed Hamiltonian and the parameter λ characterizes the strength of the perturbation. The energy eigenstates and eigenvalues are expanded in powers of λ as

$$|\psi\rangle = |\psi_0\rangle + \lambda|\psi_1\rangle + \lambda^2|\psi_2\rangle + \cdots, \tag{2}$$

$$E = E_0 + \lambda E_1 + \lambda^2 E_2 + \cdots. \tag{3}$$

Substituting these expansions into the equation $\hat{H}|\psi\rangle = E|\psi\rangle$ and collecting terms at each order of λ yield the equations for $|\psi_n\rangle$ and E_n. The zeroth-order equation is simply $\hat{H}_0|\psi_0\rangle = E_0|\psi_0\rangle$ for the unperturbed energy eigenstate and eigenvalue. The first-order equation

$$\hat{H}_1|\psi_0\rangle + \hat{H}_0|\psi_1\rangle = E_1|\psi_0\rangle + E_0|\psi_1\rangle \tag{4}$$

leads to

$$E_1 = \langle\psi_0|\hat{H}_1|\psi_0\rangle \tag{5}$$

and

$$\begin{aligned} |\psi_1\rangle &= \frac{1}{\hat{H}_0 - E_0}(E_1 - \hat{H}_1)|\psi_0\rangle \\ &= \sum_{n\neq 0} |n\rangle \frac{\langle n|\hat{H}_1|\psi_0\rangle}{E_0 - \epsilon_n}, \end{aligned} \tag{6}$$

where $|n\rangle$ and ϵ_n ($n \neq 0$) are the set of energy eigenstates and eigenvalues of the unperturbed Hamiltonian $\hat{H}_0$ excluding the ground state $|\psi_0\rangle$ and E_0. Similarly, the second-order equation

$$\hat{H}_0|\psi_2\rangle + \hat{H}_1|\psi_1\rangle = E_2|\psi_0\rangle + E_1|\psi_1\rangle + E_0|\psi_2\rangle \tag{7}$$

leads to

$$\begin{aligned} E_2 &= \langle\psi_0|\hat{H}_1 - E_1|\psi_1\rangle \\ &= \langle\psi_0|(\hat{H}_1 - E_1)\frac{1}{\hat{H}_0 - E_0}(E_1 - \hat{H}_1)|\psi_0\rangle \end{aligned} \tag{8}$$

and

$$\begin{aligned} |\psi_2\rangle &= \frac{E_2}{\hat{H}_0 - E_0}|\psi_0\rangle + \frac{1}{\hat{H}_0 - E_0}(E_1 - \hat{H}_1)|\psi_1\rangle \\ &= \sum_{n\neq 0}\left[-\frac{E_1}{(\epsilon_n - E_0)^2}\langle n|\hat{H}_1|\psi_0\rangle + \sum_{m\neq 0}\frac{\langle n|\hat{H}_1|m\rangle\langle m|\hat{H}_1|\psi_0\rangle}{(\epsilon_n - E_0)(\epsilon_m - E_0)}\right]|n\rangle. \end{aligned} \tag{9}$$

For the coupling between the molecular vibration and the optical cavity under consideration, the unperturbed ground state is $|\psi_0\rangle = |n_{\mathrm{R/P}} = 0, n_{\mathrm{c}} = 0\rangle$, i.e., no molecular vibration and no cavity photon. The coefficient c_0 in the expansions [Eqs. (10) and (11) in the main text] should therefore be close to unity. The coefficient c_2 is given

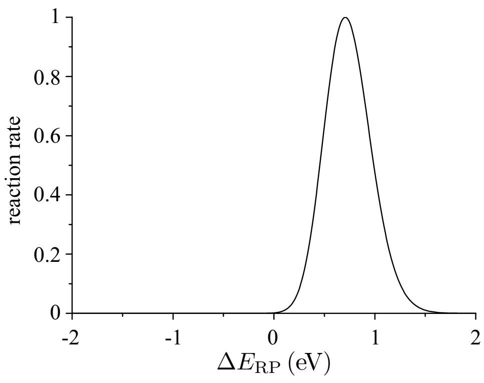

FIG. S1: Reaction rate $k(g_{\mathrm{R}}=0)$ of a bare molecule (uncoupled to the optical cavity) as a function of the energy difference ΔE_{RP} between the reactant and product states. Here the reaction rate is normalized by its maximum value at $\Delta E_{\mathrm{RP}} \simeq 0.7$ eV.

by Eq. (9) if we take $|n\rangle = |n_{\mathrm{R/P}}=2, n_{\mathrm{c}}=0\rangle$. Since there is no term in the interaction Hamiltonian [Eq. (6) in the main text] that can connect the unperturbed ground state $|n_{\mathrm{R/P}}=0, n_{\mathrm{c}}=0\rangle$to the unperturbed excited state $|n_{\mathrm{R/P}}=2, n_{\mathrm{c}}=0\rangle$, the first term on the right-hand side of Eq. (9) should vanish. On the other hand, since $\epsilon_{n,m} > E_0$, the sign of c_2 is given by that of the product $\langle n|\hat{H}_1|m\rangle\langle m|\hat{H}_1|\psi_0\rangle$. For the interaction Hamiltonian [Eq. (6) in the main text] under consideration, the unperturbed energy eigenstate $|m\rangle$ that can give a nonzero value of $\langle n|\hat{H}_1|m\rangle\langle m|\hat{H}_1|\psi_0\rangle$ is $|m\rangle = |n_{\mathrm{R/P}}=1, n_{\mathrm{c}}=1\rangle$. The operators in the interaction Hamiltonian [Eq. (6) in the main text] that give rise to nonzero values of $\langle n|\hat{H}_1|m\rangle$ and $\langle m|\hat{H}_1|\psi_0\rangle$ are $\hat{a}^{\dagger}_{\mathrm{R/P}}\hat{c}$ and $\hat{a}^{\dagger}_{\mathrm{R/P}}\hat{c}^{\dagger}$, respectively. The coefficients of these operators are both equal to $i\hbar g_{\mathrm{R/P}}$; therefore their product and in turn c_2 is a negative number.

II. REACTION RATE OF A BARE MOLECULE: ENERGY RESONANCE

Following Eqs. (8) and (9) in the main text, we numerically calculated the reaction rate $k(g_{\mathrm{R}}=0)$ of a bare molecule (uncoupled to the optical cavity) as a function of the energy difference ΔE_{RP} between the reactant and product states. Here, the parameters of the system are the same as those in the main text. The obtained result is shown in Fig. S1. It is clear that there is a strong peak at $\Delta E_{\mathrm{RP}} \simeq 0.7$ eV, which should be attributed to the effect of energy resonance. Indeed, the absolute value $|{}_{\mathrm{R}}\langle 0|n\rangle_{\mathrm{P}}|$ of the Franck-Condon factor between the vibrational ground state $|0\rangle_{\mathrm{R}}$ for the reactant configuration and a vibrational state $|n\rangle_{\mathrm{P}}$ $(n=0,1,2\cdots)$ for the product configuration is shown in Fig. S2 for the case of $\lambda_{\mathrm{v}} = 0.5$ eV (or $d_{\mathrm{RP}} \simeq 2$) considered in the main text. It is evident that $|{}_{\mathrm{R}}\langle 0|n\rangle_{\mathrm{P}}|$ is maximum near $n_0 = 2$. As a result, it can be deduced from Eq. (9) in the main text that the reaction rate would be maximum if the energy difference ΔE_{RP} satisfies $\Delta E_{\mathrm{RP}} - n_0\omega_{\mathrm{P}} \simeq \lambda$. Substituting the value of the vibrational frequency $\omega_{\mathrm{P}} = 1000\,\mathrm{cm}^{-1}$ and that of the reorganization energy $\lambda = 0.5$ eV, we find that the maximum of the reaction rate should occur at $\Delta E_{\mathrm{RP}} \simeq 0.7$ eV, as shown by the peak in Fig. S1.

On the other hand, it can be seen from Fig. 3b in the main text that the relative change of the reaction rate $k(g_{\mathrm{R}}=\omega_{\mathrm{R}})/k(g_{\mathrm{R}}=0)$ by the molecule-cavity coupling as a function of the energy difference ΔE_{RP} also shows a minimum near $\Delta E_{\mathrm{RP}} = 0.7$ eV for the case of $g_{\mathrm{P}} = 0$. This implies that if the coupling strengths g_{R} and g_{P} for the reactant and product states differ significantly from each other, the modification of the reaction rate by the molecule-cavity coupling is mainly determined by the relative shifts of energy levels in the system. The reaction rate decreases by coupling to the cavity if the rate is initially close to maximum in the bare molecular system. In contrast, it is clear from Fig. 3a that if the two coupling strengths g_{R} and g_{P} are close to each other, the relative change of the reaction rate $k(g_{\mathrm{R}}=\omega_{\mathrm{R}})/k(g_{\mathrm{R}}=0)$ by the molecule-cavity coupling is always minimum at $\Delta E_{\mathrm{RP}} = 0$, independent of the position of the resonance peak of $k(g_{\mathrm{R}}=0)$ in Fig. S1. This indicates that in this case the modification of the reaction rate is not determined by the energy shifts in the system. Instead, as shown in the main text, the modification of the reaction rate can be explained by the mixing of ground and excited states of molecular vibration in the ground state of the hybrid system, through which the Franck-Condon factor between the initial an final states of the transition is altered.

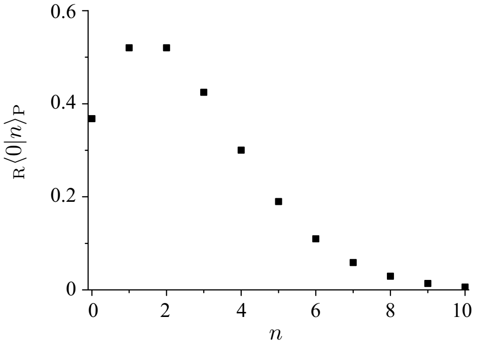

FIG. S2: Absolute value of the Franck-Condon factor $|{}_{\mathrm{R}}\langle 0|n\rangle_{\mathrm{P}}|$ between the vibrational ground state $|0\rangle_{\mathrm{R}}$ of the reactant configuration and a vibrational state $|n\rangle_{\mathrm{P}}$ $(n = 0, 1, 2\cdots)$ of the product configuration for the case of $\lambda_{\mathrm{v}} = 0.5$ eV (or $d_{\mathrm{RP}} \simeq 2$).

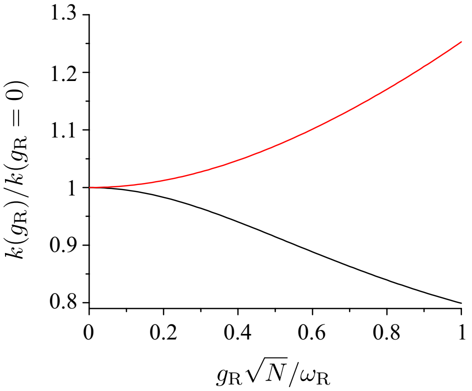

FIG. S3: Relative change $k(g_{\mathrm{R}})/k(g_{\mathrm{R}} = 0)$ of the reaction rate in a system of $N = 2$ identical molecules as a function of the collective coupling strength $g_{\mathrm{R}}\sqrt{N}$ (normalized by the vibrational frequency ω_{R}) for the two cases of $g_{\mathrm{P}} = g_{\mathrm{R}}$ (black) and $g_{\mathrm{P}} = 0$ (red). The system's parameters are the same as those in the single-molecule system.

III. MULTI-MOLECULE SYSTEM

Using Eqs. (8) and (9) in the main text, we numerically calculated the reaction rate for a system of $N = 2$ identical molecules coupled to a common cavity mode. The system's parameters are the same as those in the single-molecule system with the collective Rabi frequency (or coupling strength) $g_{\mathrm{R,P}}\sqrt{N}$ kept constant as varying from $N = 1$ to $N = 2$. The relative change $k(g_{\mathrm{R}})/k(g_{\mathrm{R}} = 0)$ of the reaction rate as a function of the normalized collective coupling strength $0 \leq g_{\mathrm{R}}\sqrt{N}/\omega_{\mathrm{R}} \leq 1$ for the two different cases of $g_{\mathrm{P}} = g_{\mathrm{R}}$ and $g_{\mathrm{P}} = 0$ are shown in Fig. S3. Here, both of the energy difference ΔE_{RP} and the detuning $\delta = \omega_{\mathrm{c}} - \omega_{\mathrm{R}}$ are set to be zero.

The dependence of the relative change of the reaction rate $k(g_{\mathrm{R}}\sqrt{N} = \omega_{\mathrm{R}})/k(g_{\mathrm{R}} = 0)$ by the molecule-cavity coupling on the energy difference ΔE_{RP} between the reactant and product states is shown in Fig. S4a for the case of $g_{\mathrm{P}} = g_{\mathrm{R}}$ and in Fig. S4b for the case of $g_{\mathrm{P}} = 0$.

The dependence of the reaction rate $k(g_{\mathrm{R}}\sqrt{N} = \omega_{\mathrm{R}})$ of the coupled molecule-cavity system on the detuning $\delta = \omega_{\mathrm{c}} - \omega_{\mathrm{R}}$ of the cavity frequency relative to the vibrational frequency is shown in Fig. S5a for the case of $g_{\mathrm{P}} = g_{\mathrm{R}}$ and in Fig. S5b for the case of $g_{\mathrm{P}} = 0$.

Compared with the results for the single-molecule system shown in Figs. 2–4 in the main text, it can be seen that the dependences of the reaction rate on the collective coupling strength $g_{\mathrm{R}}\sqrt{N}$, the energy difference ΔE_{RP} and the detuning $\delta = \omega_{\mathrm{c}} - \omega_{\mathrm{R}}$ are qualitatively similar to those of the single-molecule system. The quantitative difference between the two systems is analyzed in the main text.

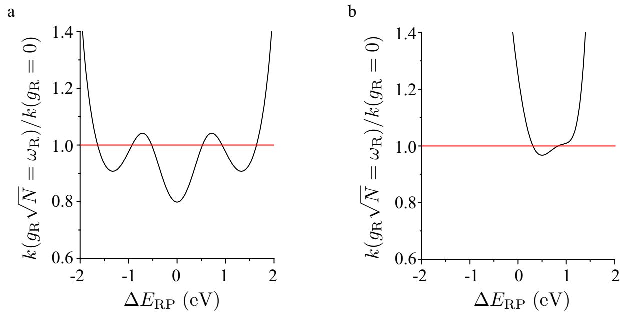

FIG. S4: Relative change of the reaction rate $k(g_{\mathrm{R}}\sqrt{N} = \omega_{\mathrm{R}})/k(g_{\mathrm{R}} = 0)$ by the molecule-cavity coupling as a function of the energy difference ΔE_{RP} between the reactant and product states for a system of $N = 2$ identical molecules. (a) $g_{\mathrm{P}} = g_{\mathrm{R}}$. (b) $g_{\mathrm{P}} = 0$. The red line shows the value of the reaction rate for the bare molecular system as a guide for the eyes.

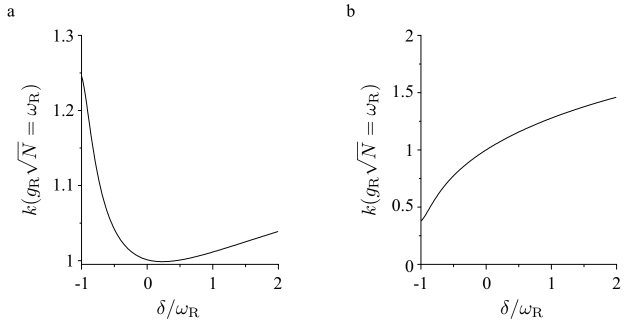

FIG. S5: Dependence of the reaction rate $k(g_{\mathrm{R}}\sqrt{N} = \omega_{\mathrm{R}})$ for a system of $N = 2$ identical molecules on the detuning $\delta = \omega_{\mathrm{c}} - \omega_{\mathrm{R}}$ of the cavity frequency relative to the molecule's vibrational frequency (normalized by ω_{R}) for the two cases of $g_{\mathrm{P}} = g_{\mathrm{R}}$ (a) and $g_{\mathrm{P}} = 0$ (b). Here, the reaction rate is normalized by its value at zero detuning $\delta = 0$.
